# A Low-Cost Method to Assess the Performance of Surface Guidance Imaging Systems at Non-Zero Couch Angles

**DOI:** 10.7759/cureus.14278

**Published:** 2021-04-03

**Authors:** Elizabeth L Covington, Richard A Popple

**Affiliations:** 1 Radiation Oncology, University of Alabama at Birmingham, Birmingham, USA

**Keywords:** sgrt, surface imaging, stereotactic radiosurgery, radiation therapy, surface-guided radiotherapy

## Abstract

A procedure is presented to assess performance at non-zero couch angles and perform routine quality assurance (QA) on surface-guided radiotherapy (SGRT) imaging systems used for stereotactic radiosurgery (SRS). A low-cost anthropomorphic phantom was used to assess the system under patient-like conditions. The phantom is embedded with a tungsten ball bearing (BB) to facilitate the use of surface imaging (SI) with concurrent megavoltage (MV) imaging to cross-compare and validate SI-reported offsets. Data analysis is done via in-house software that utilized the SGRT system’s log files for automated analysis. This procedure enables users to assess and inter-compare MV-reported offsets with their SGRT system. The analysis provides SGRT system residual error so that users are aware of inherent offsets present in addition to increases in translational offsets due to couch walkout. The procedure was validated with two commercial SGRT systems. The procedure can be used with any surface imaging system and linear accelerator system.

## Introduction

Frameless stereotactic radiosurgery (fSRS) uses an open face thermoplastic mask in place of a traditional metal frame to increase patient comfort and improve the efficiency of treatments [1-3]. The open face mask enables the use of optical surface imaging (SI) systems to monitor patients for intrafraction motion [4-6]. The use of SI for fSRS has been widely reported in the literature as an effective and efficient tool for tracking infraction patient motion [7-10].

While these systems have been widely used for intrafraction motion monitoring, there have been few reports on commissioning and quality assurance procedures for these systems [11, 12]. Surface-guided radiotherapy (SGRT) systems have also been shown to have inferior performance at non-zero couch angles [7, 9, 13-15]. In this paper, we present a detailed procedure that can be used during commissioning and routine quality assurance (QA) of surface imaging systems used for intrafraction patient monitoring during fSRS to assess performance at non-zero couch angles. This process uses a simple, low-cost phantom that is readily available. The analysis was done using in-house software that can be replicated or can be performed manually.

## Technical report

**Figure 1:**
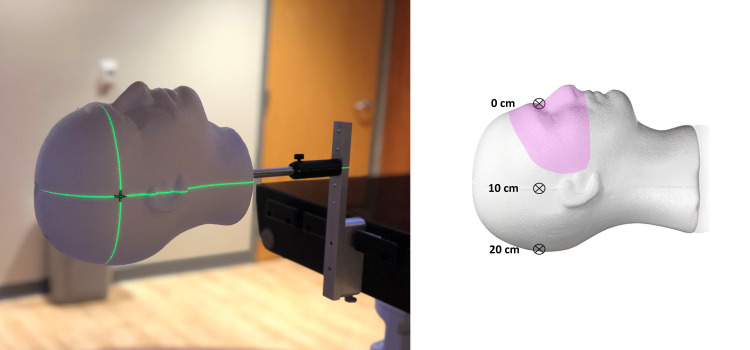
(Left) Polystyrene phantom with embedded tungsten carbide ball bearing for radiographic imaging. (Right) Representation of BB depths used for testing. All BBs were places midline with different phantoms used for each depth. A representative ROI for fSRS is shown in pink. BB: Ball bearing; ROI: Region of interest; fSRS: Frameless stereotactic radiosurgery.

An anthropomorphic phantom comprised of polystyrene (Floracraft, Ludington, MI) was embedded with a ¼” tungsten carbide ball bearing (BB) to simultaneously track the surface guided reported offsets and correlate values with those obtained with MV images obtained via the electronic portal imaging device (EPID). The BB can be placed at any location within the phantom, but was placed through the most superior surface and pushed to the midline of the phantom for routine testing. The phantom was then placed on an optical post mount (Thorlabs) and placed off the end of the couch (Figure 1) on an Edge (Varian Medical Systems, Palo Alto, CA) linear accelerator. Testing was performed for two commercial surface guidance systems, AlignRT (Vision RT, London, UK) and IDENTIFY (Varian Medical Systems, Palo Alto, CA). A region of interest (ROI) was created to be representative of an SRS ROI (e.g., the area exposed with an open face thermoplastic mask).

The phantom was positioned with the BB at isocenter via kV imaging. Once positioned, a reference surface was captured with the SGRT system to set all of the translational and rotation values to zero. Log file capture was set to be enabled for continuous recording of offsets. A treatment plan was created to enable MV imaging at couch rotations (IEC61217): 0, 22.5, 45.0, 67.5, 90.0, 270, 292.5, 315.0, and 337.5 degrees. All testing was done at a gantry angle of zero to test the system under ideal conditions without the camera pods blocked. After the images were acquired, the log file created by the surface guidance system and the DICOM files containing the MV EPID images were transferred for analysis by an in-house MATLAB (MathWorks, Natick, MA) program. The program first obtained the offset of the target ball at each table angle relative to the reference target ball position at couch 0 degrees. This step is analogous to Winston-Lutz analysis [16], where the BB walkout is determined from the variation of the BB location with respect to the collimated field. Second, the program obtained the SGRT system offsets recorded in the log file corresponding to the image acquisition times. The final step was to obtain the difference between the offsets determined from the EPID and reported by the SGRT system. This difference is the SGRT system residual error. The magnitude of the residual error was calculated by taking the root mean square of lateral and longitudinal residual error values. For example, if the MV images reported a couch walkout of 0.2 mm lateral (x) and 0.3 mm longitudinal (y), but the SGRT system reported (0.6 mm, 0.8 mm) the corresponding SGRT residual error would be (0.4 mm, 0.5 mm) with a magnitude of 0.64 mm. This test was repeated with the BB located at various depths within the phantom to determine if SI performance degrades with increasing the distance between isocenter (BB) and tracking surface.

**Figure 2:**
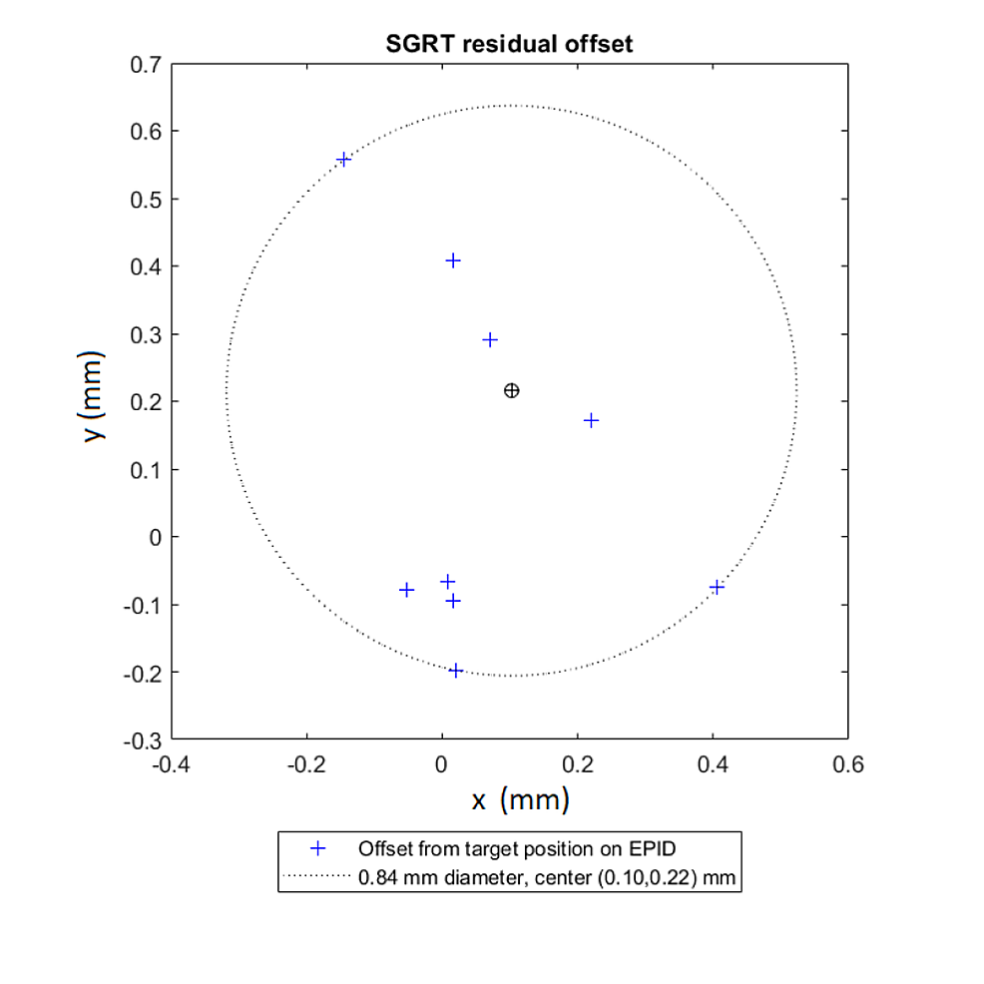
The SGRT system residual error compared to that of EPID images in the lateral (x) and longitudinal (y) direction for System A. The BB placement was at midplane depth (10 cm). SGRT: Surface guided radiotherapy; EPID: Electronic portal imaging device; BB: Ball bearing.

**Figure 3:**
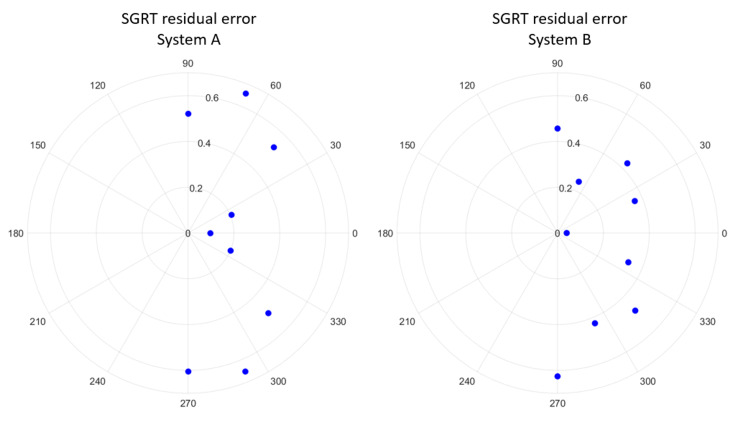
The magnitude (mm) of the SGRT system residual error as a function of couch angle (IEC61217) for each system tested on an Edge linear accelerator with the gantry at zero. BB placement was at midplane depth (10 cm) for each system. SGRT: Surface guided radiotherapy; BB: Ball bearing.

A representative data set is shown below. Figure 2 shows the residual error in the lateral (x) and longitudinal (y) direction between the EPID images and the SGRT system. The corresponding diameter of the enclosing circle is also presented. In Figure 3, the magnitude of the residual error is plotted as a function of couch angle for both systems. This is an example of the data output and the values presented are not intended to be used for performance comparison; therefore, vendor names were removed.

## Discussion

This procedure was created to assess the known issue of inferior performance of SGRT systems at non-zero couch angles [7, 9, 13-15]. The largest variation between the reference position and non-zero couch angle was found to be in the longitudinal direction after an analysis of a large patient cohort [7, 13], hence MV imaging was chosen to benchmark SGRT system performance. A portion of the SGRT system reported offsets are due to actual offset from couch walkout. To account for couch walkout, this process subtracts the MV measured couch walkout to provide the residual SGRT system error. While this study does not provide information in the vertical direction, it has been previously shown that vertical couch walkout is less than ±0.05 mm [17]. To measure residual vertical walk-out directly would require kV imaging, which is not possible at non-zero table angles because of collision between the table and the imaging system. Furthermore, the imaging arms would block the SGRT system camera pods.

While this procedure utilizes MATLAB to automate the analysis, this procedure can be easily adapted for manual analysis. For users without access to MATLAB or scripting tools, the MV images can be manually analyzed to determine the BB offset values using the treatment delivery software imaging analysis tools. When the images are taken, the user can write down the corresponding SGRT system offsets and then calculate the difference with a simple spreadsheet. To make the process less cumbersome, the number of couch angles analyzed can be reduced for routine QA after a baseline has been established during commissioning. An example worksheet is shown in Table 1.

**Table 1:** Example worksheet for calculating SGRT residual error. BB location is determined via MV imaging while phantom offsets are recorded from the surface imaging system. SGRT: Surface guided radiotherapy; BB: Ball bearing; MV: Megavoltage.

Couch Angle (°)	BB (Lat., Long.) (mm)	Phantom (Lat., Long.) (mm)	SGRT Residual Error (Lat., Long) (mm)	SGRT Residual Error Magnitude (mm)
0	0.00, 0.00	-0.05, -0.04	0.05, -0.04	0.06
90	0.10, 0.20	0.20, 0.40	-0.10, -0.20	0.22
270	0.30, 0.20	0.10, 0.60	0.20, -0.40	0.45

The automated analysis relies on the synchronization of the linear accelerator and the SI system clocks. We have observed that the clocks on both SI systems do not always stay synchronized with the treatment console; therefore, checking and correcting the time as needed before doing a test is important. Alternatively, if the surface guidance system log file contains a beam-on flag, the offset corresponding to each MV EPID image can be obtained without reliance on the timestamp.

To facilitate the use of multiple BB depths, we bought multiple phantoms to prevent confusion over which BB required analysis. Due to the low cost of the phantom, purchasing multiple phantoms was not cost-prohibitive. In a previous study [7], we utilized this technique to track the performance of our clinical SI system after software upgrades and changes to the vendor-recommended calibration workflow and an example of the residual offsets is shown at various depths. Since the intent of this technical report is to present the procedure, data for all BB depths and SI systems is not presented for brevity.

The procedure presented can be used to assess baseline SGRT system performance in addition to vendor recommended tests for commissioning and routine quality assurance. The baseline can be used to set thresholds for recalibration as part of periodic quality assurance. The residual error map can also be used to set couch angle specific action levels for reassessing patient position with radiographic imaging.

## Conclusions

We have presented a simple, low-cost procedure to assess the performance at non-zero couch angles of surface imaging systems used for stereotactic radiosurgery treatments. The procedure uses a plastic foam anthropomorphic head that is inexpensive and widely available. Combined with tungsten BBs, the phantom can be used to correlate the difference in MV image and the SGRT system reported offsets at various couch angles. Analysis can be done via software or by manual analysis. This procedure enables clinics to determine any discrepancies between the on-board imaging system and the SGRT system and determine a baseline performance for the SGRT system at non-zero couch angles under ideal conditions. This procedure can be used for routine quality assurance in addition to vendor recommended procedures.
